# Association between Regular Use of Analgesics before Cancer Diagnosis and Occurrence of Mood Disorders

**DOI:** 10.3390/nursrep14030136

**Published:** 2024-07-24

**Authors:** Hyun Sook Oh, Subin Noh, Hwa Jeong Seo

**Affiliations:** 1 Department of Applied Statistics, School of Social Science, Gachon University, Seongnam 461-701, Republic of Korea; hoh@gachon.ac.kr; 2 Global Healthcare Research Institute, Gachon University, Seongnam 461-701, Republic of Korea; nsbin3@gachon.ac.kr; 3 Medical Informatics and Health Technology (MiT), Department of Healthcare Industry Management, Gachon University, 1342 Seongnamdaero, Sujeong-gu, Seongnam 461-701, Republic of Korea

**Keywords:** cancer survivor, depression, anxiety, cohort study, prescription

## Abstract

We aimed to determine the relationship between the use of analgesics prescribed for pain management and the onset and progression of mood disorders using a large-scale cohort database. We calculated hazard ratios (HR) with 95% confidence intervals (CI) for patient risk of developing mood disorders based on age, income, health-related variables, disease history, Charlson comorbidity index, and analgesics prescription behavior (Models 1–3). Additionally, we determined the risk of mood disorder occurrence by age group (Model 4) using a proportional hazards regression model. The age- and income-adjusted HR (Model 1) was 1.8275. The age-, income-, BMI-, and physical-activity-adjusted HR (Model 2) was 1.882. The fully adjusted HR (Model 3) was 1.698. Compared with no analgesic use, nonregular use (HR = 1.386) and regular use (HR = 1.698) was associated with a higher risk of mood disorders. Among patients older than 50 years, those who participated in physical activity (less than five days) had a lower risk of mood disorders than those who did not. This suggests that it may be useful for preventing mood disorders in older cancer survivors. A high risk of comorbidities and regular use of analgesics are risk factors for developing mood disorders. Therefore, our results suggest that cancer survivors with a high risk of comorbidities and a history of regular analgesic use should undergo careful psychiatric consultation.

## 1. Introduction

Cancer survivors with improved 5-year relative survival rates have been reported to contract several chronic diseases while undergoing treatment. Scholars emphasize the need for and importance of the management of secondary cancer prevention and screening, comorbidities, lifestyle habits, and psychological problems [1]. In cases where the management of diseases other than cancer is neglected, death may result from comorbidities rather than cancer [2].

Patients with cancer experience various sociopsychological problems, such as mental distress, during cancer diagnosis, treatment, and return to daily life [3]. Distress reduces treatment compliance and quality of life and, in the long term, causes increased cortisol secretion and decreased immunity, which can directly affect survival and recurrence [4,5]. A meta-analysis of prospective studies revealed that patients with cancer having depressive symptoms had a 25% higher mortality rate and those with major depression had a 39% higher mortality rate than those without symptoms [6]. Research indicates that the frequent use of psychotropic drugs can lead to early death in patients with cancer. Accordingly, the psychosocial management of such patients is considered as important as cancer treatment [7].

Research evidence supporting the involvement of chronic pain and inflammatory conditions in the development and progression of mood disorders [8] has enhanced interest in drug repurposing for the management of highly treatment-resistant mood disorders [9]. Reports indicate that inflammation is mainly associated with somatic symptoms of depression, whereas social risk factors are related to cognitive and emotional symptoms. Acetaminophen and nonsteroidal anti-inflammatory drugs (NSAIDs), which are commonly prescribed for pain management, have potential chemopreventive effects on mood disorder symptoms and risks [10,11]. Accordingly, studies are being conducted to determine the relationship between the use of analgesics and the development and progression of mood disorders. In a randomized controlled trial involving 62 people, acetaminophen has been demonstrated to relieve physical as well as psychological pain [12]. Furthermore, in a randomized controlled study of 1497 osteoarthritis patients, NSAIDs such as ibuprofen, Naproxen, and Celebrex have exhibited a positive effect on reducing mood disorder symptoms compared to placebo (*p* = 0.039) [13]. However, in a large-scale cohort study of 22,564 Spanish university graduates, regular use of NSAIDs was not significantly associated with a reduced risk of mood disorders (HR: 1.33, 95% CI: 0.59–2.99) [14]. Additionally, in a cohort study of 14,992 seniors aged 64 or older suffering from cancer and osteoarthritis, the use of analgesics was identified as a major predictor of depression [15].

Cancer patients are one of the risk groups with a 3 to 5 times higher risk of developing mood disorders than the general population, highlighting the need for priority screening and prevention [16]. Therefore, based on a large cohort population, we sought to determine how regular use of analgesics before cancer diagnosis affects the occurrence of mood disorders such as depression and anxiety. Its purpose is to provide evidence to improve the quality of life of survivors by analyzing the correlation between the use of analgesics and the occurrence of mood disorders, such as depression and anxiety.

## 2. Materials and Methods

### 2.1. Study Design and Data Collection

The National Health Insurance Service–National Sample Cohort (NHIS-NSC) is a population-based retrospective cohort associated with a 2.2% representative sample of South Korean citizens [17,18].

This study examines the effects of drug use following drug exposure in patients with newly diagnosed cancer. In this study, sampling was performed as follows: *Malignant neoplasms* of the female reproductive system (gynecological cancer; malignant neoplasms of the breast, ovary, cervix and corpus, vulva and vagina, and other unspecified female reproductive organs) were defined as the presence of the same C code more than once (*n* = 9136) [19].

Patients who were male (*n* = 67) and aged <20 years (*n* = 79) at the index date were excluded. Furthermore, patients diagnosed with cancer in 2002 (*n* = 1789) and whose death (autopsy) was confirmed following cancer diagnosis (*n* = 9) were excluded from the study. Patients with no history of health examination before cancer diagnosis (*n* = 3347) and those with a history of mood disorders (F code appearance = at least once, antianxiety medication prescription ≥ 30 days, or antidepressant prescription ≥ 30 days) prior to cancer diagnosis were excluded (*n* = 155). Finally, 3628 participants were included in the analysis.

### 2.2. Criteria and Definitions

**Figure 1:**
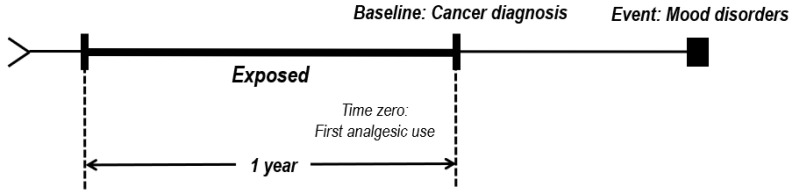
Study design–associated follow-up from cohort entry to event occurrence.

This study examined age (<50 and ≥50 years), income quantile (low, 1st–3rd quantile; middle, 4th–6th quantile; and high, 7th–10th quantile) [20], physical activity duration (never, <5 days, and ≥5 days), body mass index (BMI) (<25 and ≥25), disease history (hypertension and dyslipidemia), Charlson comorbidity index (CCI), and analgesics prescription behavior. The group with mood disorders included the following members: Participants for whom an F code (mental and behavioral disorder) appeared more than once in the main illness variable in the medical treatment database or patients who had been prescribed antidepressants (N06A) and antianxiety drugs (N05B) for more than 15 days within the observation period [21,22,23]. Further, the participants’ health behavior, disease history, CCI, and analgesic use (regularity of use) were calculated based on their medical records up to 1-year follow-up before cancer diagnosis (Figure 1).

CCI is a comorbidity measurement tool that predicts the 1-year mortality rate of patients with breast cancer based on medical record survey data. Based on a patient’s pre-diagnosis history, we weighed and included 17 comorbidities (Myocardial infarction, Congestive heart failure, Peripheral vascular disease, Cerebrovascular disease, Dementia, Peptic ulcer disease, Renal disease, Moderate or severe liver disease, etc.). Further, we used an algorithm conforming to ICD-10 to calculate the CCI score based on the 1-year observation period before the patient’s first health examination (excluding AIDS) [24]. The analysis considered hypertension and dyslipidemia, which are not included in CCI but are common chronic diseases.

### 2.3. Assessment of First-Step Analgesic Use

This study examined the effectiveness of participants’ medication compliance (regularity) based on the World Health Organization’s three-step ladder analgesics, which are indicated for pain caused by various conditions such as anxiety/depression (mood disorder), diabetes, high blood pressure, and obesity. The first-step analgesics considered were acetaminophen, aspirin, piroxicam, diclofenac, celecoxib, ibuprofen, naproxen, mefenamic acid, ketoprofen, and dexibuprofen.

Analgesic use was determined by extracting the prescription history for first-step analgesics over a period of 1 year from baseline. Detailed information on use was collected when the drug was used on a “regular” basis (i.e., prescription for twice a month for >6 months, prescription for >7 days per month for 2 consecutive months, or 90 or more prescriptions in 365 days). The participants who had irregularly used a drug 7 days per month were considered “nonregular” users. Finally, those who had never used the drug were categorized as “never” users [25].

### 2.4. Statistical Analysis

The impact of differences in demographics, health behaviors, disease history, CCI, and first-step analgesic use on the occurrence of mood disorders was examined using the chi-square test. We calculated hazard ratios (HRs) with 95% confidence intervals (CIs) for patient’s risk of developing mood disorders. We used 3 models with increasing degrees of adjustment to account for potential confounding factors at baseline. Model 1 was adjusted for age (<50 or ≥50 years) and income (low, middle, and high). Model 2 was further adjusted for body mass index (<25 or ≥25), and physical activity (never, <5 times per week, and ≥5 times per week). Model 3 was further adjusted for hypertension (yes or no), dyslipidemia (yes or no), and Charlson comorbidity index (0 or ≥1). Additionally, we determined the risk of occurrence of mood disorders by age group (Model 4) using a proportional hazards regression model. All the statistical analyses were performed using the statistical package R, version 4.2 (R Core Team, Vienna, Austria).

## 3. Results

### 3.1. Participant Characteristics

Table 1 presents the participants’ characteristics. Whereas 611 (16.84%) patients were in the group with mood disorders, 3017 (83.16%) were in the group without mood disorders. 

In the group without mood disorders, 1439 (47.70%) participants were under 50 years of age and 1578 (52.30%) were more than 50 years of age; 1533 (50.81%) recorded high income, 908 (30.10%) middle income, and 675 (22.37%) low income. Further, 1347 women (44.65%) never exercised. Disease history variables indicate that 892 women (29.57%) had hypertension and 814 (26.98%) had dyslipidemia. Further, 1565 (51.87%) and 1452 (48.13%) participants had CCI scores of 0 and ≥1, respectively. Regarding analgesic use, 1692 (56.08%), 893 (29.60%), and 432 (14.32%) participants were nonregular users, nonusers, and regular users, respectively.

In the group with mood disorders, 213 (34.86%) and 398 (65.14%) were under and more than 50 years of age, respectively; further, 331 (54.17%) had high, 163 (26.68%) middle, and 117 (19.15%) low incomes, respectively. Whereas 331 women (54.17%) never engaged in physical activity, 178 (20.61%) engaged in less than 5 days and 102 (20.61%) always engaged in physical activity. Regarding disease history variables, 226 women (36.99%) had hypertension, and 168 (27.50%) had dyslipidemia. Further, 257 (42.06%) and 354 (57.94%) participants had CCI scores of 0 and ≥1, respectively. Finally, 358 (58.59%) patients were nonregular users, 122 (19.97%) were nonusers, and 131 (21.44%) were regular users of analgesics.

### 3.2. Risk Factors Associated with Mood Disorder Development in Cancer Survivors

Cox regression analysis was performed to determine the effects of analgesic use (regularity) on the time taken for mood disorder incidence among the 3628 cancer-surviving participants. For Model 1, the age and income-adjusted HR was 1.875 (95% CI: 1.458–2.411). Further, for Model 2, the age, income, BMI, and physical activity-adjusted HR was 1.882 (95% CI: 1.461–2.424). Finally, the fully adjusted HR for Model 3 was 1.698 (95% CI: 1.301–2.215) (Table 2). 

**Figure 2:**
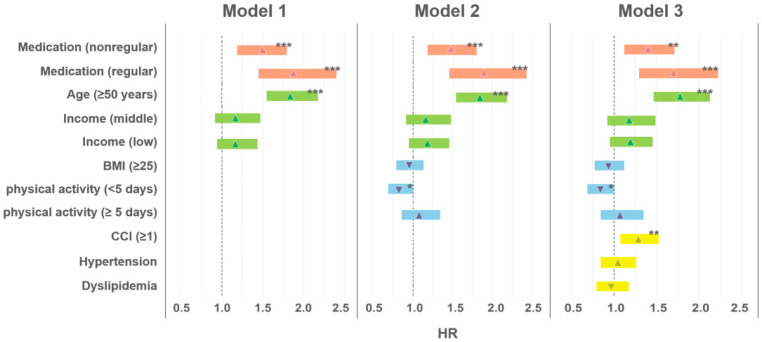
Impact of analgesic use on the development of mood disorders: medication prescribing behavior related to analgesic use (orange), demographics (green), health examination–related factors (blue), and possible confounding factors (the severity of comorbidities; yellow); BMI, body mass index; CCI, Charlson comorbidity index; HR, hazard ratio. * *p* > 0.1, ** *p* > 0.05, *** *p* > 0.001.

Figure 2 depicts the HR for each variable adjusted for Models 1, 2, and 3. Model 1 is the HR of analgesics when adjusted for age and income. HR (*p* < 0.001, HR = 1.838) was higher in people over 50 years of age. Model 2 is adjusted for BMI and physical activity. At this time, HR (*p* < 0.001, HR = 1.837) was high in those over 50 years of age, and HR (*p* < 0.1, HR = 0.832) was low in those who exercised (less than 5 days). Model 3 is adjusted for CCI, hypertension, and dyslipidemia. Age and physical activity exhibited significant results, and CCI (more than 1) demonstrated a high HR (*p* < 0.05, HR = 1.279).

### 3.3. Association between Mood Disorder and Analgesic Use by Age

Among the participants in the <50-year age group, compared with those having a CCI score = 0, those with a CCI score ≥ 1 had a significantly higher risk of mood disorders (HR = 1.309, 95% CI: 0.991–1.730, *p* < 0.1). Compared with no analgesic use, nonregular use was associated with a higher risk of mood disorders (HR = 1.582, 95% CI: 1.135–2.207, *p* = 0.007), and regular use was associated with a higher risk of mood disorders (HR = 2.634, 95% CI: 1.658–4.185, *p* < 0.001).

Among the patients in the ≥50-year age group, the ones who participated in physical activity (less than 5 days) had a lower risk of mood disorders (HR = 0.719, 95% CI: 0.565–0915, *p* = 0.007) than those who did not participate. Further, regular analgesic use (HR = 1.418, 95% CI: 1.026–1.960, *p* = 0.034) was associated with a significantly higher risk of mood disorders compared to irregular use (Table 3).

## 4. Discussion

This study examined the association between pre-diagnosis analgesic use and distress risk in cancer survivors. The study’s main findings are as follows:

First, the older the cancer survivor (≥50 years), the higher the risk of occurrence of mood disorders. Consistent with earlier studies [26], the current study associated older age with an increased risk of mood disorders. The average age of menopause in Korean women is 50 years old [27]. The finding that the risk of mood disorders is higher for those over 50 years of age compared to those below 50 years is believed to be associated with menopause [28,29].

Second, physical activity was confirmed to lower the risk of mood disorder development in participants. In particular, adequate (moderate) activity was associated with a reduced risk of mood disorder development in women over 50 years of age [30]. Physical activity is known to be one of the modifiable health behaviors that can alleviate depressive and anxiety symptoms in clinical and paraclinical settings. Several large-scale studies and meta-analyses indicate that physical activity is associated with a reduction in total depression symptom scores both cross-sectionally and longitudinally [31,32]. Although the exact relationship between physical activity and mood disorders is not clear, the former is believed to protect against depression and anxiety symptoms [33]. It is recommended that cancer survivors exercise moderately three to four days a week. For women over the age of 50, moderate (3–4 days a week) rather than excessive exercise (5 or more days a week) can lower the risk of developing mood disorders.

Third, participants with high comorbidities and disease severity have a high risk of occurrence of mood disorders. A positive relationship was confirmed between comorbidities and the risk of mood disorder development [34]. Therefore, cancer survivors with these characteristics require appropriate psychological interventions and management.

Mood disorders are complex conditions caused by various factors, including stress, endocrine abnormalities, and genetic predispositions [35]. Patients with *severe illnesses* like cancer are at a high risk of mood disorders due to the psychological stress caused by the diagnosis or treatment process or the physical pain caused by the disease itself.

Fourth, the pre-diagnosis use of WHO’s first-step analgesics was found to be associated with a high risk of mood disorders. Epidemiologic evidence indicates that proinflammatory conditions increase the risk of colorectal cancer, whereas the chronic use of NSAIDs potentially reduces the risk [36]. Kohler et al. clarify that NSAIDs, which have anti-inflammatory effects, can help prevent and treat mood disorders by reducing systemic inflammation [10]. The inhibition of cyclooxygenase (COX), which is targeted by analgesics such as NSAIDs, increases oxidative stress in the brain and impairs antioxidant activity and mitochondrial function. Therefore, it may worsen the biological mechanisms affecting the development of mood disorders [37]. This emphasizes the importance of developing treatment strategies targeting various pathways, rather than a single pathway, involved in the development of mood disorders.

Further, Dewall et al. [12] report that acetaminophen reduces anxiety. However, some other studies indicate that it blunts sensitivity to both negative and positive stimuli [38]. Acetaminophen and NSAIDs have excellent safety and versatility and are used to relieve various symptoms; therefore, it is common to take both drugs together [39]. Polypharmacy, which is the simultaneous use of multiple drugs, can cause interactions among drugs and result in unexpected side effects [40]. A meta-analysis including 19 studies found that polypharmacy was associated with an increase in the risk of mood disorders (odds ratio, OR: 1.73, 95% CI: 1.39–2.14, *p* < 0.001) [41]. These results emphasize the importance of carefully interpreting the relationship between the use of analgesics and the development of mood disorders because this relationship stems from various causes.

In an analysis targeting patients with cancer, osteoarthritis [15], acute coronary artery disease [42], and patients admitted to the intensive care unit [43], the use of analgesics increased the risk of developing mood disorders. Mood disorders arise from a variety of causes, including stress, endocrine abnormalities, and genetic predisposition, and are the result of complex interactions [35]. For patients with critical illnesses such as cancer, the use of analgesics may not be sufficient to significantly reduce the risk of mood disorders due to the psychological stress resulting from the diagnosis or treatment process and the physical pain caused by the disease itself.

In this study, we focused on the history of regular use of prescribed analgesics to increase compliance with pain treatment. It was confirmed that the use of analgesics that were prescribed pre-diagnosis for patients with cancer having higher pain levels than the general population was associated with the post-diagnosis development of mood disorders such as depression and anxiety.

The incidence of cancer is particularly high in women with high-risk comorbidities, such as diabetes and hypertension [44]. Regular users of first-step analgesics to counter comorbid conditions, including these chronic diseases, are at high risk of developing mood disorders, even after their cancer diagnosis. Additionally, it can have a negative impact on cancer prognosis [6,45,46], which suggests the need for systematic management.

This study has the following limitations: First, the NHIS-NSC claims data used as an analysis data source comprised sample data extracted from a stratified sample of the entire population, rather than a specific disease, and the study failed to classify in detail the malignant neoplasms of the female reproductive system. Since the NHIS-NSC cohort masks only the malignant neoplasms of reproductive organs (breast cancer, cervical and corpus cancer, ovarian cancer, vulva and vaginal cancer, and other unspecified malignant neoplasms of reproductive organs), the corresponding carcinoma could not be specified. Second, although the study analyzed the long-term use of analgesics, it did not consider over-the-counter use. Third, some earlier studies [47] report the effects of painkillers, particularly Celecoxib and Aspirin, in suppressing mood disorders. However, the current study could not clearly confirm the effects of these drugs due to sample size limitations caused by domestic reimbursement standards. Nevertheless, despite these limitations, this study is meaningful because it classified patients with gynecological cancer into specific age groups (based on the average age at menopause) and analyzed the relationship between the use of painkillers and the development of mood disorders.

Collaboration between nurses, physicians, and psychiatrists is essential in developing individualized care plans for cancer survivors at high risk of mood disorders due to regular analgesic use and comorbidities. Nurses can facilitate multidisciplinary discussions to integrate psychiatric consultations into survivorship care plans and ensure holistic management of patient’s physical and mental health needs. Nurses play a crucial role in educating cancer survivors about the potential risks associated with regular analgesic use, particularly in relation to mood disorders. Therefore, nurses should incorporate thorough assessment and ongoing monitoring of cancer survivors’ analgesic use patterns and mood status as part of routine care.

## 5. Conclusions

Cancer survivors over 50 years of age had a higher risk of developing mood disorders than those below 50. In particular, the risk of regular analgesic use was more significant in younger (<50 years) than older (>50 years) women. The current study recommends that cancer survivors ≥50 years of age exercise moderately (less than five days a week), rather than excessively (five or more days a week), to lower the risk of developing mood disorders. This suggests that it may be a useful strategy for preventing mood disorders in older cancer survivors.

A high risk of comorbidities and regular use of analgesics have been identified as risk factors for developing mood disorders. Therefore, our results suggest that cancer survivors with a high risk of comorbidities and a history of regular analgesic use should undergo careful psychiatric consultation.

The regular use of analgesics such as acetaminophen and NSAIDs was considered a risk factor for developing mood disorders; however, this relationship depends on the analgesics’ mechanism of action. Moreover, additional studies are required to overcome the limitations of this study.

## Figures and Tables

**Table 1:** Baseline characteristics of the study population.

Variable		Mood Disorders				*x* ^2^	*p*
		No (*n* = 3017)		Yes (*n* = 611)			
		N	%	N	%		
Age							
	<50	1439	47.70	213	34.86	33.237	<0.001
	≥50	1578	52.30	398	65.14		
Income							
	Low (1~3)	675	22.37	117	19.15	3.541	0.170
	Middle (4~6)	908	30.10	163	26.68		
	High (7~10)	1533	50.81	331	54.17		
BMI (Body Mass Index)							
	<25	2147	71.16	426	69.72	0.445	0.505
	≥25	870	28.84	185	30.28		
Physical activity							
	Never	1347	44.65	331	54.17	18.779	<0.001
	<5	1088	36.06	178	29.13		
	≥5	582	19.29	102	16.69		
CCI (Charlson comorbidity index)							
	0	1565	51.87	257	42.06	19.171	<0.001
	≥1	1452	48.13	354	57.94		
Hypertension							
	No	2125	70.43	385	63.01	12.785	<0.001
	Yes	892	29.57	226	36.99		
Dyslipidemia							
	No	2203	73.02	443	72.50	0.045	0.832
	Yes	814	26.98	168	27.50		
Analgesics use							
	Never	893	29.60	122	19.97	34.020	<0.001
	Non-regular	1692	56.08	358	58.59		
	Regular	432	14.32	131	21.44		

**Table 2:** Hazard ratios associated with mood disorder development in a cohort.

	Model 1			Model 2			Model 3		
	HR ^(1)^	95% CI ^(2)^	*p*	HR	95% CI	*p*	HR	95% CI	*p*
Analgesics use									
Never	1.000			1.000			1.000		
Non-regular	1.464	1.191–1.799	<0.001	1.465	1.192–1.800	<0.001	1.386	1.123–1.710	0.002
Regular	1.875	1.458–2.411	<0.001	1.882	1.461–2.424	<0.001	1.698	1.301–2.215	<0.001

^(1)^ HR: hazard ratio, ^(2)^ CI: confidence interval. Model 1: adjusted for age (<50, ≥50) and income. Model 2: Further adjusted for body mass index (<25, ≥25) physical activity (never, <5 days, and ≥5 days). Model 3: Further adjusted for Charlson comorbidity index (0, ≥1), hypertension (no or yes), dyslipidemia (no or yes).

**Table 3:** Mood disorder development risk by age group.

Model 4			<50			≥50	
		HR ^(1)^	95% CI ^(2)^	*p*	HR	95% CI	*p*
Income							
	Low (1~3)	1.000			1.000		
	Middle (4~6)	1.070	0.710–1.612	0.746	1.210	0.902–1.622	0.203
	High (7~10)	1.143	0.794–1.644	0.472	1.199	0.924–1.555	0.173
BMI (Body Mass Index)							
	<25	1.000			1.000		
	≥25	0.770	0.530–1.120	0.171	0.982	0.795–1.215	0.870
Physical activity							
	Never	1.000			1.000		
	<5	1.037	0.776–1.385	0.806	0.719	0.565–0.915	0.007 **
	≥5	0.889	0.555–1.424	0.625	1.135	0.876–1.470	0.340
CCI (Charlson comorbidity index)							
	0	1.000			1.000		
	≥1	1.309	0.991–1.730	0.058 *	1.279	1.029–1.589	0.026
Hypertension							
	No	1.000			1.000		
	Yes	1.295	0.888–1.889	0.180	0.992	0.801–1.229	0.944
Dyslipidemia							
	No	1.000			1.000		
	Yes	1.024	0.667–1.573	0.912	0.973	0.787–1.203	0.799
Analgesics use							
	Never	1.000			1.000		
	Non-regular	1.582	1.135–2.207	0.007 **	1.242	0.947–1.629	0.117
	Regular	2.634	1.658–4.185	<0.001 ***	1.418	1.026–1.960	0.034 **

^(1)^ HR: Hazard ratio, ^(2)^ CI: confidence interval. * *p* > 0.1, ** *p* > 0.05, *** *p* > 0.001.

## Data Availability

Data are available upon reasonable request to the corresponding author.

## Public Involvement Statement

No public involvement in any aspect of this research.

## Guidelines and Standards Statement

This manuscript was drafted against the Strengthening the Reporting of Observational Studies in Epidemiology (STROBE) guidelines.
